# Ticagrelor versus clopidogrel for recurrent myocardial infarction: An outcomes-based agreement

**DOI:** 10.1016/j.rcsop.2023.100347

**Published:** 2023-10-13

**Authors:** Samuel K. Peasah, Yan Huang, John Venditto, Robert Brekosky, Daniel Belletti, Vanessa Campbell, Chronis Manolis, Chester B. Good

**Affiliations:** a Value-Based Pharmacy Initiatives, Center for High Value HealthCare, UPMC Health Plan, India; b AstraZeneca Pharmaceuticals, UK; c Pharmacy Services, UPMC Health Plan, India

**Keywords:** Ticagrelor, Clopidogrel, Myocardial infarction, Acute coronary syndrome, Outcomes research

## Abstract

**Background:**

Outcomes-based agreements (OBA) are performance-based risk-sharing agreements between manufacturers and payers which provide the opportunity for collection and evaluation of real-world outcomes to supplement clinical trials.

**Objectives:**

To describe an OBA comparing ticagrelor to clopidogrel in patients admitted with acute coronary syndrome (ACS) and proportion of recurrent myocardial infarction (MI) in a real-world setting.

**Methods:**

Commercial (CM) and Medicare (MC) insurance patients of a large regional health plan, who presented with ACS and were prescribed either ticagrelor or clopidogrel were prospectively analyzed. The cohort consisted of adults (18–85 years) discharged between January 1, 2019, and December 31, 2020, who were adherent to the study medications, within the confines of the OBA. The primary outcome of interest was the proportion of recurrent MI hospitalizations within one year of discharge.

**Results:**

There were 500 patients who met inclusion criteria in the ticagrelor cohort and 648 in the clopidogrel cohort. The mean age of patients in the ticagrelor cohort was 61.5 ± 10.5 years old and 66.5 ± 10.2 years in the clopidogrel cohort. The proportion of patients with type 2 diabetes, hypertension, or a history of congestive heart failure at baseline in the ticagrelor cohort was 31%, 85%, 14% respectively, and 43%, 90%, and 32% respectively in the clopidogrel cohort. The overall proportion of hospitalization for recurrent MI was 1.00% in the ticagrelor and 3.13% in the clopidogrel cohorts. In the follow-up propensity-matched analysis, although recurrent MI hospitalization was higher in the clopidogrel cohort (1.69% vs 1.21%) it was not statistically significant (*p*-value 0.5242).

**Conclusion:**

Patients presenting with ACS and treated with ticagrelor had a lower rate of hospitalization for recurrent MI compared to patients treated with clopidogrel cohort within the confines of an OBA in a real-world setting.

## 1 Introduction

Demand for value has been at the forefront of the Centers for Medicare & Medicaid Services (CMS) value-purchasing programs and other pay-for-performance initiatives.^1^ Outcomes-based agreement (OBA) (alternatively called value-based contracting) is a payment arrangement between manufacturers and payers. It is designed to provide payers an opportunity to earn additional rebate or reimbursement if the drug underperforms on an agreed outcome measure in a real-world setting. These performance-based risk-sharing agreements may increase access to innovative medications for patients, lead to more favorable formulary position for manufacturers' products, and provide potential additional discount for payers.^1^, ^2^, ^3^ Although these agreements are increasing in the United States and many other countries, they have not had the impact on payment reforms as experts had hoped. However, these agreements provide the opportunity to obtain and analyze real-world use of these often-newer products to supplement clinical trial data.

There are multiple concerns and/or barriers with OBA contracts including a lack of transparency, and difficulty in measuring meaningful outcomes (using less expensive methods such as through claims datasets). Additionally, there are concerns of rare disease conditions with lack of alternative drug options, and acute conditions with short follow-up periods or limited financial impact.^3^ Knowledge or details of these contracts are usually confidential and not divulged to the public and outcomes or findings are often not published especially in peer-reviewed journals.^3^, ^4^, ^5^

A large regional health plan entered an OBA with the manufacturer of ticagrelor (Brilinta), a P2Y12 inhibitor, in 2018 and the outcome of interest was recurrent myocardial infarction (MI) after an acute coronary syndrome (ACS) discharge. The margins for success in the OBA were based upon the results of the Platelet Inhibition and Patient Outcomes (PLATO) trial in which ticagrelor was shown to be superior to clopidogrel in the composite primary endpoint of vascular death causes, MI, or stroke among patients presenting with ACS^6^.

The results of the OBA are presented in this study. The OBA sought to determine whether treatment with ticagrelor instead of clopidogrel in patients admitted with ACS would reduce the proportion of recurrent MI in a real-world setting. Manufacturers, payers, and managed care organizations may find this study useful in assessing the value of OBAs and the value proposition of these newer but more costly pharmaceuticals.

## 2 Methods

This was a prospective study of patients of a large regional health plan, who presented with ACS and were prescribed either ticagrelor or clopidogrel within 45 days of discharge from January 1, 2019, to December 31, 2020. Study inclusion required, age 18–85 years at discharge, enrollment in either a commercial (CM) or Medicare (MC) line of business, and adherence to the study medications. To be adherent, the patient must have been on the study medication ≥80% of the time over a year or by date of recurrent MI from the index fill.

Patients with ACS at discharge were identified using ICD-10 codes for UA (120.0), STEMI (121.xx, 122.xx), and NSTEMI (121.4). Medication adherence was measured using proportion of days covered (PDC) from the index fill to either an MI event, or end of the follow-up period (maximum 365 days). Exclusion criteria, based on the OBA, included: Patients who were not continuously enrolled over the study period, patients who switched between the study medications, and patients who had recurrent MI within 14 days of initiating study medications.

The outcome was the proportion of recurrent MI (ICD-10 codes-121.0,121.01, 121.02, 121.09, 121.1, 121.11, 121.19, 121.2, 121.21, 121.29, 121.3, 121.4) hospitalization occurring between 14- and 365-days post discharge. The primary outcome of interest was the proportion of recurrent MI hospitalizations in patients prescribed ticagrelor compared to those prescribed clopidogrel. There were no adjustments made for differences in baseline characteristics in this real-world analysis for the OBA Model of the analysis, in keeping with the OBA. Although not included in the OBA, to add scientific rigor, cohorts were propensity-matched in a follow-up analysis (Propensity Model), controlling for demographic differences including age, sex, insurance type, Charlson comorbidity index (CCI), medical condition (type 2 diabetes mellitus (T2DM), hypertension, congestive heart failure (CHF)), prior percutaneous cutaneous intervention (PCI) within 12 months of event (hospitalization) date, and prior use of direct oral anticoagulants (DOAC) within 12 months of event date. Then, a simple logistic regression of the matched cohorts (2 × 2) of recurrent MI (yes/no) vs. study medication (ticagrelor/clopidogrel) was conducted.

Descriptive statistics and logistic regression were performed using SAS 9.4 and statistical significance was defined at the 0.05 level.

## 3 Results

### 3.1 OBA model

**Fig. 1:**
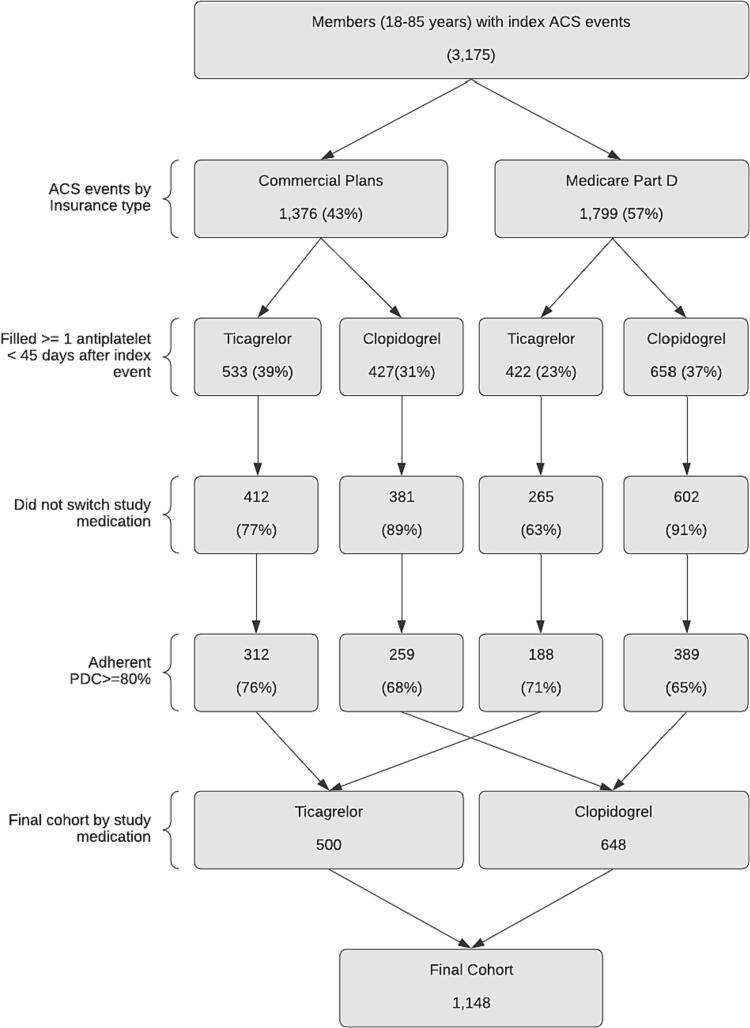
Flowchart of ticagrelor and clopidogrel cohorts by line of business.

Of the 3175 index ACS admissions within the study period, 975 (30.7%) filled at least one prescription of ticagrelor within 45 days of discharge and 1085 (34.2%) filled at least one prescription of clopidogrel. The final adherent sample consisted of 500 patients in the ticagrelor cohort and 648 patients in the clopidogrel cohort. (Fig. 1).

#### 3.1.1 Baseline characteristics

A higher proportion of the clopidogrel cohort were from MC (60%) compared to 38% from the ticagrelor cohort which likely contributed to the difference in mean ages (clopidogrel: 66.5 ± 10.5 years, ticagrelor: 61.5 ± 10.2). Similarly, there were higher proportions of patients with T2DM (43% vs 31%) and CHF (33% vs. 14%) in the clopidogrel cohort compared to the ticagrelor cohort. Additionally, a higher proportion of patients received a direct oral anticoagulant (DOAC) within 12 months of the index admission (12% vs. 3%) in the clopidogrel compared to the ticagrelor cohort (Table 1).

**Table 1:** Baseline characteristics of members prescribed ticagrelor or clopidogrel after ACS discharges.

Patient Baseline Characteristics	Subtype	Ticagrelor (*n* = 498)	Clopidogrel (*n* = 640)
Age, Mean (Std)		61.54 (10.16)	66.52 (10.53)
Gender, N (%)	Male Female	369 (74.10) 129 (25.90)	404 (63.13) 236 (36.87)
Insurance Type, N (%)	Commercial	311 (62.45)	257 (40.16)
	Medicare Part D	187 (37.55)	383 (59.84)
Charlson Comorbidity Index		3.00 (3.00)	4.00 (3.00)
Disease conditions	Hyperlipidemia, N (%)	444 (89.16)	582 (90.94)
	Type 2 Diabetes, N (%)	152 (30.52)	272 (42.50)
	Hypertension, N (%)	424 (85.14)	575 (90.00)
	Congestive Heart Failure, N (%)	71 (14.26)	206 (32.19)
Had Percutaneous Coronary Intervention	12 months prior to Index RX Date, N (%)	8 (1.61)	33 (5.16)
Had Direct Oral Anticoagulants (DOAC)	12 months prior to Index RX Date, N (%)	14 (2.81)	74 (11.56)
Days from Anchor Discharge to Index RX, Mean (Std)		6.97 (10.66)	5.64 (9.26)
Type of Acute Coronary Syndrome (ACS) at admission	NSTEMI N (%)	231 (46.4)	462 (72.2)
	STEMI N (%)	266 (53.4)	175 (27.3)
	Unstable Angina N (%)	1 (0.2)	3 (0.5)

The mean CCI was 3 ± 3 in the ticagrelor cohort and 4 ± 3 in the clopidogrel cohort. The two cohorts differed with respect to type of ACS at discharge: 46% NSTEMI, 53% STEMI, and 0.2% UA in the ticagrelor cohort, and 72% NSTEMI, 27% STEMI, and 0.5% UA in the clopidogrel cohort. PCI during the index admission was performed in 93% of the patients prescribed ticagrelor compared to 90% of patients prescribed clopidogrel. (Table 1).

#### 3.1.2 Outcomes

**Fig. 2:**
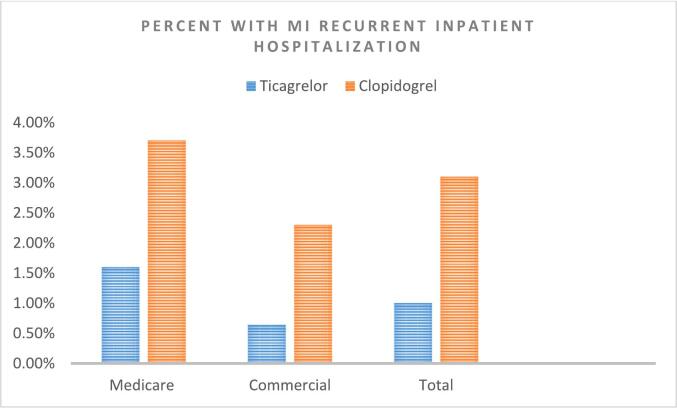
Proportion of Recurrent Myocardial Infarction Hospitalizations by Cohort and Insurance Type.

The overall proportion of hospitalization for recurrent MI was 1.00% in the ticagrelor cohort (CM: 0.64% and MC:1.60%) and 3.13% in the clopidogrel cohort (CM: 2.33% and MC: 3.66%) [Fig. 2]. The proportion of hospitalization for recurrent MI in the subgroup of patients who underwent PCI during index hospitalization was similar to the overall group; 1.00% in the patients prescribed ticagrelor, and 3.13% in the patients prescribed clopidogrel.

### 3.2 Propensity model

The number of people that propensity 1:1 matched were 360 each. The two cohorts were evenly matched and were not statistically different from each other. The significant differences between the OBA and Propensity Models were that the clopidogrel cohort in the Propensity Model was younger (62.7 vs. 66.5 yrs.), with a lower proportion of patients with T2DM (33% vs. 43%), CHF (16% vs. 32%), and prior PCI (1% vs. 5%).

The simple logistic regression found no statistical difference in recurrent MI between the two cohorts (ticagrelor 0.56 vs. clopidogrel 0.83, *p* = 0.5242).

## 4 Discussion

OBAs evaluate use of innovative drugs in a real-world setting, tying their performance to financial incentives. Despite the popularity of OBAs, there are few reports in the literature that document their results. In this report, an OBA is described that was based on the comparative effectiveness of a branded product (ticagrelor) with a less expensive comparator drug (clopidogrel). The chosen outcome was based on a comparison of the proportion of recurrent hospitalizations for MI among patients prescribed either clopidogrel or ticagrelor after presenting with ACS.

Randomization to ticagrelor in the PLATO trial resulted in a significant absolute risk reduction in recurrent MI, (5.8% vs 6.9% for ticagrelor and clopidogrel respectively and a 16% relative risk reduction).^6^ In patients in this OBA, there were fewer admissions for recurrent MI in the ticagrelor cohort than the clopidogrel cohort (1.00% vs. 3.13%) and the unadjusted risk of hospitalization for recurrent MI was reduced by 67% which was greater than the relative risk reduction seen in PLATO. This large reduction could reflect greater acuity in treatment cohorts consisting of only adherent patients without adjustment for baseline characteristics, as well as the relatively small number of patients included in the OBA. The MI reduction was 33% in the adjusted Propensity Model, although not statistically significant. It is important to note that the OBA study was not powered to detect statistical differences between the 2 cohorts. In the RCT PLATO study, the authors estimated that they needed 1078 events (death, MI, and stroke) for a power of 90% to detect differences between the two cohorts. They recruited 18, 624 patients for the study and our propensity-matched cohorts were 360 patients in each cohort.

In a study of ticagrelor vs. clopidogrel for MI recurrent admissions of 5 distinct health plan OBAs of 31,946 patients using pooled data from 2015 to 2018, the recurrent rates were 4.10% vs. 2.03% in favor of ticagrelor (RRR = 50.5% range [24–65.7%]). These results were consistent with our study findings including higher MI recurrent rates in MC than in CM.^7^ Conversely, there are other reports in real-world settings that showed no statistically significant differences between ticagrelor and clopidogrel MI readmissions^8^^,^^9^.

In a recent large retrospective cohort study of patients with ACS discharged on ticagrelor or clopidogrel between January 1, 2012, and September 30, 2019, using the Optum Clinformatics database, ticagrelor significantly reduced the rate of hospitalization for MI compared to clopidogrel. The ticagrelor and clopidogrel cohorts were balanced by propensity score matching 1:3 for demographic and clinical characteristics. Hospitalization for MI was significantly lower in the ticagrelor compared to the clopidogrel cohort (2.22 versus 3.52 per 100 patient-years; 36.8% relative risk reduction [RRR]; *P* < 0.0001).^10^

Our data demonstrate that patients prescribed ticagrelor differed from those prescribed clopidogrel with respect to baseline demographics and comorbidities. A higher proportion of patients with ACS STEMI were prescribed ticagrelor rather than clopidogrel (53% vs. 27%). This use of P2Y12i was consistent with the 2021 ACC/AHA revascularization guideline recommendation (2a (B-R) that it is reasonable to use ticagrelor in preference to clopidogrel to reduce ischemic events in patients with ACS undergoing PCI^11^. This recommendation was based on the results of the PLATO trial in which ticagrelor significantly reduced overall major adverse cardiac events (MACE) and the risk for subsequent MI and CV death compared to clopidogrel in patients with ACS. The benefit of ticagrelor over clopidogrel in PLATO was most evident in higher ischemic risk patients with NSTEMI-ACS, diabetes, chronic kidney disease, and peripheral artery disease. However, patients at greater risk of subsequent ischemic events in the current study, including older patients with more comorbidities such as diabetes and heart failure, were prescribed clopidogrel more often than ticagrelor. In addition, patients with NSTEMI-ACS, who have greater long-term risk of recurrent CV events than those presenting with STEMI-ACS were prescribed clopidogrel far more often than ticagrelor. In general, the choice of clopidogrel over ticagrelor in older Medicare patients may be driven by both bleeding risk and cost considerations. However, recent evidence indicates that the use of ticagrelor monotherapy as an option to dual-antiplatelet therapy (DAPT) may mitigate the increased risk of bleeding associated with ticagrelor DAPT.^12^^,^^13^

Interestingly, in the present study, adherence rates were higher for ticagrelor than for clopidogrel in both the CM and MC cohorts. This may have been a result of the reduced out-of--pocket expense for ticagrelor in the MC cohort as part of the OBA, combined with efforts of the health plan to improve adherence in high-risk patients. Ticagrelor, being a brand medication, is higher priced but the cost-effectiveness of ticagrelor over clopidogrel has been established in several global studies.^14^, ^15^, ^16^, ^17^

Findings of this study as well as other pooled studies bring attention to OBAs. These agreements serve not only as a viable payment model, especially for newer medications with high uncertain effectiveness beyond RCTs, but also provide other payers data to potentially decide formulary placements of these medications. OBAs designed with more scientific rigor are likely to add to the comparative effectiveness research findings of these medications despite the legal and implementation constraints.

### 4.1 Limitations

This OBA was an observational study and as such subject to selection bias preventing any causality conclusions. Consequently, although this OBA was designed to mimic the PLATO RCT study eligibility criteria, it did not include adjustments of baseline characteristics, and was not powered to make any conclusions. However, results reflect characteristics of patients prescribed ticagrelor or clopidogrel post ACS-discharge in a large regional health plan.

## 5 Conclusion

In an OBA of ticagrelor vs clopidogrel for preventing MI recurrent admissions among adherent patients in a large regional health plan, the event rate was lower in the ticagrelor cohort than the clopidogrel cohort. Moreover, reporting the findings of this OBA will provide prescribers the opportunity to assess other prescribers use of clopidogrel vs ticagrelor for patients with ACS, and also for other third-party payers, the potential role of OBA in formulary decisions or as an alternative payment model.

### 5.1 Summary

Findings of an outcomes-based agreement that compared recurrent myocardial infarction rates between ticagrelor and clopidogrel among patients with acute coronary syndrome.

## Funding

This work was not funded.

## CRediT authorship contribution statement

**Samuel K. Peasah:** Writing – original draft, Formal analysis, Writing – review & editing. **Yan Huang:** Formal analysis, Writing – review & editing. **John Venditto:** Writing – review & editing. **Robert Brekosky:** Conceptualization, Writing – review & editing. **Daniel Belletti:** Conceptualization, Writing – review & editing. **Vanessa Campbell:** Writing – review & editing. **Chronis Manolis:** Conceptualization, Writing – review & editing. **Chester B. Good:** Supervision, Conceptualization, Writing – review & editing.

## Declaration of Competing Interest

The authors are employees of either UPMC Health Plan or AstraZeneca. This project is a result of an outcomes-based agreement between the two organizations.

The authors report of no conflict of interest beyond being employees.
